# Laparoscopic Treatment of Intrauterine Fallopian Tube Incarceration

**DOI:** 10.1155/2013/205957

**Published:** 2013-04-30

**Authors:** William Kondo, Rafael Frederico Bruns, Marcelo Chemin Nicola, Reitan Ribeiro, Carlos Henrique Trippia, Monica Tessmann Zomer

**Affiliations:** ^1^Department of Gynecology, Sugisawa Medical Center, Curitiba, PR, Brazil; ^2^Department of Gynecology, Vita Batel Hospital, Curitiba, PR, Brazil; ^3^Department of Radiology, Fetalmed, Curitiba, PR, Brazil; ^4^Department of Gynecology, Nossa Senhora de Fátima Maternity Hospital, Curitiba, PR, Brazil; ^5^Department of Radiology, Roentgen Diagnóstico Institute, Curitiba, PR, Brazil

## Abstract

Herniation of the pelvic structures into the uterine cavity (appendix vermiformis, small bowel, omentum, or fallopian tube) may occur after uterine perforation. In this paper, we describe one case of intrauterine fallopian tube incarceration treated by means of laparoscopic surgery.

## 1. Introduction

Uterine perforation during curettage is a potentially dangerous complication but may go unrecognized on many occasions [1]. Herniation of the pelvic structures into the uterine cavity, such as the appendix vermiformis, small bowel, omentum or fallopian tube, occurring after uterine perforation has been described in the medical literature but is very rare [1–5]. In this paper, we describe one case of intrauterine fallopian tube incarceration treated by means of laparoscopic surgery.

## 2. A Case Presentation

**Figure 1:**
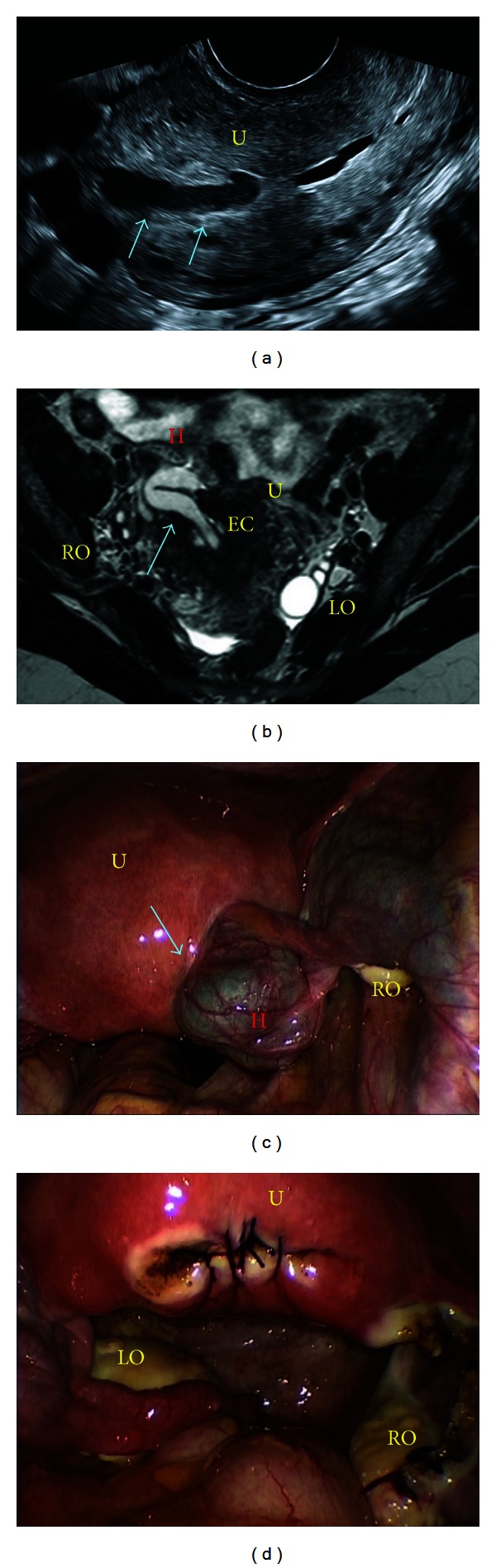
(a) Transvaginal ultrasound showing a hypoechoic structure (blue arrows) within the uterus (U). (b) Pelvic MRI demonstrating a herniation (blue arrow) of the right hydrosalpinx (H) through the uterine wall (U) going up to the endometrial cavity (EC). Both the right (RO) and the left (LO) ovaries were normal. (c) Laparoscopic visualization of the pelvic cavity: the hydrosalpinx (H) is incarcerated (blue arrow) in the uterus (U), and the right ovary (RO) is normal. (d) Final aspect of the procedure: uterus (U), left ovary (LO), and right ovary (RO).

A 22-year-old woman, gravida 2 para 2, came to our office complaining about pelvic pain and amenorrhea since her vaginal delivery. The symptoms of pain were intermittent, but they worsened in the last 3 days before she came to our service including persistent, cramping abdominal pain, and mild abdominal distension. Eleven months ago, she had her second vaginal delivery complicated by retained placenta. The placenta was delivered in multiple fragments followed by sharp curettage. Then, she presented postpartum hemorrhage requiring another curettage of the uterus. On physical examination, the abdominal examination was unremarkable. Gynecologic examination revealed a tender uterus with no adnexal abnormalities. Transvaginal ultrasound (Figure 1(a)) revealed a hypoechoic, irregular tissue within the endometrial cavity. The ovaries were normal. Pelvic MRI (Figure 1(b)) demonstrated a right hydrosalpinx that “infiltrated” the uterine fundus, extending to the endometrial cavity. A diagnostic laparoscopy (Figure 1(c)) was indicated, and during the procedure, the right fallopian tube was found to be adhered to the uterine fundus. The right ovary and the left adnexae were normal. The tube was progressively freed from the uterine wall. A right salpingectomy was conducted because the patient did not want to have any more pregnancies. The uterine wall defect was repaired in multiple layers using caprofyl (poliglecaprone 25) zero (Figure 1(d)). The patient was discharged 12 hours after the procedure.

## 3. Discussion

Fallopian tube prolapse through the uterus may occur as a consequence of uterine perforation [1–5]. The correct diagnosis of intrauterine fallopian tube incarceration is difficult because of nonspecific clinical manifestations. However, a preoperative diagnosis can be made with the proper use of imaging techniques [3].

The ultrasonographic signs of an intrauterine digestive or fallopian tube incarceration are typical: a hyperechoic tubular structure within the myometrium [2]. In the instance of an intestinal incarceration, one may observe the layers of the intestinal wall, the presence of peristaltic movements, hypo- or hyperechoic content, and/or hydro-air levels [2]. In the case presented here, the dilated fallopian tube (hydrosalpinx) presented on transvaginal ultrasound as a hypoechoic tubular structure within the uterine wall. The MRI may be helpful to confirm the images obtained from the transvaginal ultrasound. 

Whenever intrauterine incarceration of an intra-abdominal structure is suspected, the surgical treatment is indicated. The hysteroscopy may be performed before the laparoscopy to visualize and partially release the intrauterine incarceration [2]. The laparoscopic procedure will confirm the diagnosis and allow for the release of the incarcerated structure from the myometrium. If the patient desires to become pregnant, the fallopian tube may be gently extracted from the uterine wall, and, depending on the aspect of the fimbrial portion, a salpingoplasty may be performed [2] or not [1, 4]. Hysterosalpingography and hysteroscopy should be performed 3 months after the surgical procedure to confirm the uterine wall healing and the absence of synechia and to check the tubal patency [2]. Two cases of spontaneous pregnancy after surgical treatment of intrauterine fallopian tube incarceration have been described in the literature [2]. Women with no desire for pregnancy may be surgically managed by means of a salpingectomy [3, 5]. 

To the best of our knowledge, only a few cases of intrauterine fallopian tube incarceration have been described in the medical literature. Although rare, this entity should be kept in mind especially in those women with a previous history of dilatation and curettage with uterine perforation.
